# Endoscopic nasal dacryocystorhinostomy: results and advantages over the external approach

**DOI:** 10.1016/S1808-8694(15)31335-5

**Published:** 2015-10-20

**Authors:** Daniel Salgado Küpper, Ricardo Cassiano Demarco, Renato Resende, Wilma Terezinha Anselmo-Lima, Fabiana Cardoso P. Valera, Iracema Moribe

**Affiliations:** ^1^ Specialist by SBORL, Assistant Physician; ^2^ Professor, Assistant Physician; ^3^ Specialist in ORL, Resident physician; ^4^ Full Professor, Associate Professor; ^5^ Professor, Assistant Physician; ^6^ Ph.D., Professor, Assistant Physician

**Keywords:** dacryocystorhinostomy, surgery of the lachrymal pathways, endoscopic technique

## Abstract

**D**acryocystorhinostomy (DCR) is a procedure used to create a lachrymal drainage pathway into the nasal cavity in order to reestablish the permanent drainage of a previously obstructed excretory system. **Aim**: to report our results obtained with endoscopic DCR technique, describing its advantages and disadvantages **Study design**: Historic cohort. **Material and Method**: we retrospectively analyzed thirty-two dacryocystorhinostomies performed at the Otorhinolaryngology Discipline from March 2002 to January 2004 on patients with post-lachrymal sac obstruction confirmed by dacryocystorhinography (DCG). In all cases, the patients were submitted to probing with Crawford probe. **Results**: surgery was bilateral in ten of the twenty-two analyzed patients, totaling thirty-two procedures, twenty-nine of which were primary surgeries and three revision procedures after unsuccessful external DCR. Our success rate was 79.12%. **Conclusions**: endoscopic DCR proved to be a safe and low morbidity technique, which also avoids facial scars and maintains the mechanism of the lachrymal pump, with results similar to those obtained with external DCR.

## INTRODUCTION

Dacryocystorhinostomy (DCR) consists of creating a lachrymal drainage pathway to the nasal cavity to restore permanent drainage of the previously obstructed excreting system, through an opening normally made at the level of the lachrymal bone^1^. Classically, it has been performed by ophthalmologists using an external approach. However, thanks to the use of endoscopes, endonasal dacryocystorhinostomy has been proven to be a safe and effective surgical technique to solve low lachrymal obstructions. It has become an alternative to the approach of lachrymal pathways, owing to low morbidity and results equivalent to the conventional external surgical approach.

Lachrymal pathway obstruction may occur at any point of the tract and for surgical purposes, it is classified as pre-saccal (common canaliculus), saccal or post-saccal (nasolachrymal duct). Thus, knowledge of the anatomy of lachrymal pathways is essential for the understanding of the pathological processes and to be able to correctly perform diagnostic and therapeutic procedures.

Lachrymal points mark the onset of the lachrymal drainage system, followed by the upper and lower canaliculi, which are fused and form the common canaliculus, measuring about 1 to 2mm long, which emerges anteriorly into the lachrymal sac. After it, the tear is pumped, via nasolachrymal duct, up to being exteriorized into the nasal cavity together with the lower meatus, about 1.5cm posterior to the margin of the lower concha head. The lachrymal sac is recovered by nasal mucosa and by a bone wall (anterior to the maxillary frontal process and posteriorly to the lachrymal bone), located anteriorly to the unciform process. The upper border is placed above the insertion of the middle concha and the inferior border close to the upper portion of the lower concha. The lachrymal sac has about 10mm of vertical extension, out of which about 3-5 mm are above the insertion of the common canaliculus. There are studies that show that the lachrymal sac may extend up to 8mm above the insertion of the middle concha1., 2.. The main valve system of the nasolachrymal duct can be found in the junction of the lachrymal sac (Rossenmuller) and in the opening of the lower meatus (Heisner), preventing lachrymal reflux. These are the sites of congenital obstruction of the lachrymal system.

In addition to congenital affections, other obstructive causes are considered, such as iatrogenic causes (post-facial surgeries, for example), traumatic, lithiasis and acute infections that progress to stenosis or fibrosis of lachrymal pathways^2^. However, most of the etiological factors that lead to obstruction are idiopathic, more frequent in Caucasian women in the 5th and 6th decades of life. The most frequent complaints are ocular affections, such as epiphora, lachrymal sac recurrent infections, chronic drainage and persistent purulent secretion through the lachrymal points and cutaneous fistulization.

The suspicion of obstruction may be confirmed by Jones test, which consists of the instillation of fluorescein in the eye and the endoscopic observation of nasal drainage with staining in the lower meatus (negative test for total obstruction). This method only detects obstruction and does not define its level, requiring to that end DCG that can predict the surgical diagnosis according to the contrast applied to the lachrymal sac. Normal size or increased lachrymal sac is predictive of good results in endoscopic DCR, whereas reduced lachrymal sac suggests fibrosis and higher likelihood of post-surgical stenosis^3^. Partial contrast or no contrast of common canaliculus suggests pre-saccal obstruction, better approached by conjunctivodacryocystorhinostomy.

Our purpose was to report the experience and the results of our service with the endoscopic technique of dacryocystorhinostomy, showing the advantages and disadvantages of this technique in relation to the external access.

## MATERIAL AND METHOD

We analyzed 32 procedures of dacryocystorhinostomy carried out by the Ambulatory of Lachrymal Pathways, Sector for Rhinosinusology, Hospital das Clínicas, Medical School, Ribeirão Preto - USP in 22 patients with manifestations of epiphora or repetitive dachryocystitis and radiographic diagnosis of post-saccal obstruction. The cases were operated on via endoscopy, with minimum postoperative follow-up of four months and maximum of 2 years.

The diagnosis of lachrymal obstruction was confirmed through the dripping test or infusion of fluorescein in the conjunctiva or lachrymal canaliculus (Jones test) without observation of stained nasal drainage. The level of obstruction was defined by dacryocystography. Out of 22 patients, 12 presented unilateral obstruction and 10 had bilateral obstruction.

The employed surgical technique was conducted with the patient under general anesthesia, dorsal decubitus and elevated bed rest, to have less venous pressure and less bleeding. We conducted infiltration of the lateral wall with lidocaine solution at 1% and adrenaline at 1:80000, from the projection of the lachrymal bone to the unciform apophysis. Under visualization with 0º endoscope we made an incision of mucoperiostal flap at the level of the lachrymal fossa, 1-cm in extension, with posterior limit close to the unciform process, which was detected and resected. Thus, we visualized and removed the bone medial wall of the lachrymal sac through drilling or resection with scope up to its complete exposure. The lachrymal sac can be identified using optic fiber transilumination or by introducing a probe through the canaliculi, normally observed with angled endoscope of 30 or 45º. We made an incision of the lachrymal sac in the inferior portion of the upper margin with a scythe or electrocautery. We preferred to use a Crawford probe in all cases to ensure permeability of new drainage route. In those with associated nasal pathology, especially with obstructive septal deformities, we made concomitant corrections to improve surgical access. In case of associated septoplasty, we placed splints without nasal packing.

We prescribed oral and eye drops antibiotics and corticoids to minimize the formation of granuloma or stenosis of the opening. Post-surgical follow up was performed weekly to remove crusts, granulation tissue and possible synechia up to complete local epithelialization.

## RESULTS

The age of patients submitted to endoscopic dacryocystorhinostomy ranged from 11 to 69 years, mean age of 30.1 years. Out of 22 analyzed patients, the surgery was bilateral in 10, totaling 32 procedures. Out of them, 29 were primary surgeries and there were 3 cases of revision procedures (failure of previous dacryocystorhinostomy by external access).

**Figures:**
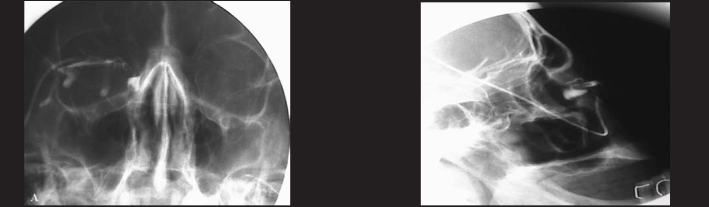
Dachryocistography showing obstruction of post-saccal lachrymal sac obstruction on the right. A) frontal view; B) lateral view.

**Table 1:** Surgical results of endoscopic dacryocystorhinostomy.

CASE	AGE (YEAR)	TYPE	SIDE	ASSOCIATED SURGERIES
1	18	External access revision	Right	Septoplasty
2	22	Primary	Bilateral	Septoplasty
3	18	Primary	Bilateral	No
4	60	Primary	Bilateral	No
5	41	External access revision	Right	No
6	18	Primary	Bilateral	No
7	11	Primary	Bilateral	No
8	42	Primary	Bilateral	No
9	61	Primary	Left	No
10	67	Primary	Right	No
11	37	Primary	Left	Septoplasty
12	48	Primary	Bilateral	Septoplasty
13	69	External access revision	Bilateral	Septoplasty
14	66	Primary	Left	No
15	50	Primary	Left	No
16	39	Primary	Left	No
17	64	Primary	Bilateral	Septoplasty
18	72	Primary	Bilateral	No
19	13	Primary	Right	Septoplasty
20	51	Primary	Right	Septoplasty
21	53	Primary	Left	No
22	65	Primary	Right	Anterior ethmoidectomy and right maxillary medium meatotomy

Postoperative pain was mild, and we used analgesics to control it. We did not observe complications such as epistaxis, subcutaneous emphysema, damage to orbital structures or visual affections. One of the patients presented mild periorbital ecchymosis without visual impairment and progressed with spontaneous resolution. Another patient presented synechia between the medium concha and the lateral wall.

We reached total resolution of manifestations in 79.12% (25 surgeries). However, seven cases progressed with reobstruction, without complete resolution of clinical symptoms and they were included among those not considered as surgical success. One of the patients that did not reach improvement had severe facial trauma with severe deformity as etiology of lachrymal obstruction, presenting technical difficulty to identify the intranasal anatomical structures. All revision cases previously submitted to external approach surgery had resolution of epiphora and repetitive dachryocystitis when accessed via endoscopy.

Nine patients were submitted to other endoscopic procedure to correct associated pathologies, eight septoplasties for obstructive septal deformity and one patient was submitted to anterior ethmoidectomy and medium meatotomy owing to chronic maxillo-ethmoidal rhinosinusitis.

## DISCUSSION

Endoscopic dacryocystorhinostomy has been demonstrated to be a safe and low morbidity technique with efficacy that ranged in the literature from 80 to 90%3., 4., 5., 6., 7., 8.. Other authors reported success over 90%3., 9.. External surgery presents similar results of about 90%10., 11.. Success rate of our sample was of about 80% even when the team of residents performed it, similarly to the literature reports.

As to surgical technique, we observed that to succeed in the surgery, we have to use wide opening of medial wall of lachrymal sac, minimizing the risk of restenosis^8^. Another factor to be considered for surgical success is regular cleaning of the nasal cavity, performed endoscopically, to prevent formation of synechiae or granulomas that hinder permeability of lachrymal fistula, especially after removal of lachrymal probe.

The use of a probe is quite controversial. Some authors use it as a routine, whereas others use it in selected cases such as in severe lachrymals sac fibrosis or in cases of revision surgery7., 9., 12.. Other authors have already suggested that the probe increases the rate of recurrence owing to higher likelihood of promoting formation of granulation tissue with posterior stenosis of the fistula1., 12., 13.. However, in our series, we decided to go for the placement of a silicone probe in all patients, to maintain permeability of lachrymal pathway up to end of healing period, which was removed three to six months after the surgery.

The choice of indication between external or endoscopic approach depends on the service where the patient is being seen, because normally external access is performed by Ophthalmologists whereas endoscopic access is preferred by Otorhinolaryngologists. The advantages of endoscopic DCR are quite a few compared to external approach: absence of external scars, maintenance of a lachrymal pump system through the orbicularis muscle (which is sectioned in the external approach), less bleeding, in addition to being easier in revision procedures, because the lachrymal sac is already exposed owing to the previous osteotomy. The possibility of performing repairs in the same surgical time for the association with nasal pathologies or those that are prone to recurrence (septal deformity, rhinosinusitis or synechiae of previous surgeries, patients with history or predisposition to formation of keloids), emphasize the endonasal indication3., 14., 15.. Endonasal approach may be preferred also in cases of acute dachryocystitis resistant to antibiotics.

In our series, the incidence of complications using endoscopic access was low, even though the literature reports the likelihood of ocular-orbital lesions, especially of orbital fat exposure, orbital hematoma, subcutaneous emphysema and severe epistaxis7., 9.. In our sample, we did not have severe complications, only periorbital ecchymosis without visual impairment and of spontaneous resolution.

## CONCLUSION

We concluded that the endoscopic technique of DCR is a safe, effective, low complication technique that yields good esthetical-functional results and that it shows a success rate similar to that of external approach when performed by experienced surgeons.
